# A prospective pilot study of home monitoring in adults with cystic fibrosis (HOME-CF): protocol for a randomised controlled trial

**DOI:** 10.1186/s12890-017-0366-x

**Published:** 2017-01-23

**Authors:** Jocelyn Choyce, Karen L. Shaw, Alice J. Sitch, Hema Mistry, Joanna L. Whitehouse, Edward F. Nash

**Affiliations:** 1 West Midlands Adult CF Centre, Heart of England NHS Foundation Trust, Bordesley Green East, Birmingham, B9 5SS UK; 2 Institute of Applied Health Research, College of Medical and Dental Sciences, University of Birmingham, Edgbaston, Birmingham B15 2TT UK; 3 Division of Health Sciences, Warwick Medical School, University of Warwick, Coventry, CV4 7AL UK

**Keywords:** Cystic fibrosis, Home monitoring, Pulmonary exacerbations, Health related quality of life, Health economics

## Abstract

**Background:**

Home monitoring has the potential to detect early pulmonary exacerbations in people with cystic fibrosis (CF), with consequent improvements in health outcomes and healthcare associated costs. This study aims to assess the effects of home monitoring on hospital admissions, quality of life, antibiotic requirements, exacerbation frequency, lung function, nutritional outcomes, anxiety, depression, costs and health outcomes, as well as the qualitative effects on the patient experience.

**Methods:**

This randomised controlled mixed-methods trial aims to recruit 100 adults with CF cared for in one large regional CF centre. Participants are randomly allocated 1:1 to the intervention group (twice-weekly home monitoring of symptoms measured by the Cystic Fibrosis Respiratory Symptom Diary – Chronic Respiratory Infection Symptom Score (CFRSD-CRISS) and Forced Expiratory Volume in one second (FEV_1_)) or a control group (routine clinical care) for the 12-month study period. Measurements are recorded at study visits at baseline, 3, 6, 9 and 12 months. Spirometry, body weight, co-morbidities, medications, hospital inpatient days, courses of antibiotics (oral and intravenous), pulmonary exacerbations (defined by the modified Fuchs criteria) are recorded at each study visit. Health status, capability and health economics are measured at each study visit by the Hospital Anxiety and Depression Scale (HADS), the ICEpop CAPability measure for Adults (ICECAP-A), EuroQol 5 dimensions (EQ-5D-5L) questionnaire and an adapted resource use questionnaire. The patient experience is assessed by semi-structured qualitative interviews at baseline and 12 months.

**Discussion:**

Results from this study will help to determine the effect of home monitoring on inpatient bed days and quality of life in adults with CF, as well as other relevant health and health economic outcomes.

**Trial registration:**

This study protocol is registered with Clinicaltrials.gov (NCT02994706), date registered 16^th^ July 2014

## Background

Cystic fibrosis (CF) is the most common fatal inherited condition in Caucasians, affecting more than 10,000 people in the UK [1] and at least 70,000 people worldwide [2]. CF is caused by abnormal function of the cystic fibrosis transmembrane conductance regulator (CFTR), a complex protein which functions primarily as a chloride channel, regulating movement of ions and fluid across epithelial surfaces [3]. CFTR dysfunction causes epithelial secretions to be abnormally dehydrated and viscous, resulting in damage to several organs including the lungs, pancreas, intestines and liver. Within the lungs, chronic infection and inflammation results in progressive lung disease and premature death due to respiratory failure. Survival in CF has dramatically improved over the last 40 years due to factors including improvements in antibiotic therapy, better nutrition and the development of centres devoted to the provision of specialised CF care. As a result, CF has changed from a predominantly paediatric disease to the current situation where the majority of individuals with CF survive into adulthood. The median predicted survival in the UK is currently in excess of 45 years of age [1] and that of people with CF born in the year 2000 is predicted to be in excess of 50 years of age [4].

People with CF often experience a burden of symptoms on a daily basis, typically including: cough, sputum production, wheeze, breathlessness, chest tightness and fever [5]. When patients develop worsening symptoms, they are advised to contact their CF multidisciplinary team (MDT) and may be diagnosed with a pulmonary exacerbation. Pulmonary exacerbations are typically treated with antibiotics targeted at the bacteria that cause chronic infection in that individual patient. However, symptoms of a pulmonary exacerbation do not usually develop suddenly, but instead worsen slowly over the course of several days to weeks. Patients presenting with less severe symptoms are more likely to receive a course of oral antibiotics, but as the exacerbation worsens the patient may require intravenous antibiotics. Intuitively, it would be expected that if the patient were diagnosed with a pulmonary exacerbation earlier, with milder symptoms, the requirement for intravenous antibiotics would be less.

Anecdotally we know that some patients are able to perceive a change in their symptoms better than others. Some realise that they are developing an exacerbation early on, whereas others present when they have already become more severely unwell. Additionally, patients may know that they are developing an exacerbation, but are unable to come to clinic due to other commitments or due to a lack of clinic capacity. In either case, we suspect that if we were able to diagnose the exacerbation at an earlier stage we may be more likely to be able to treat it successfully with a course of oral antibiotics, rather than intravenous antibiotics. Additionally, in our experience if the patient is less unwell they are more likely to be able to receive intravenous antibiotics at home, rather than requiring admission. This could potentially be beneficial to the patient, preventing the complications of a more severe infection and could also reduce healthcare expenditure by averting a hospital admission.

Several previous studies have assessed the effect of telehealth or home monitoring in people with CF, but the vast majority of these studies have been small feasibility studies with limited external validity [6–13]. A randomised pilot study in CF patients demonstrated that home monitoring was able to detect more pulmonary exacerbations than standard care [14]. A large randomised controlled study of home monitoring is currently ongoing in the USA, assessing the primary outcome of change in forced expiratory volume in 1 s (FEV_1_) over a 12-month period [15]. Secondary outcome measures include the effect of home monitoring on CF respiratory symptom scores, pulmonary exacerbations, health-related quality of life, treatment burden, change in prevalence of resistant strains of bacteria and adverse events. There are no cost-effectiveness analyses included in this study and no qualitative assessment of the patient experience.

The primary aims of the current study are to (1) determine whether home monitoring is effective compared with routine care in reducing hospital inpatient bed days; and (2) assess whether this results in differences in health-related quality of life over a 12 months period in adults with CF. We hypothesise that participants randomly allocated to home monitoring will require fewer hospital inpatient bed days and that they will have better health-related quality of life compared with those receiving routine care.

## Methods/Design

### Study design

This single-centre, non-blinded, randomised controlled mixed-methods trial will be conducted at West Midlands Adult CF Centre, Heart of England NHS Foundation Trust, Birmingham, UK.

### Participants

#### Inclusion/exclusion criteria

To be eligible for enrolment, participants must be able to give informed consent, be aged ≥ 18 years and have a diagnosis of CF confirmed by clinical characteristics, sweat test and/or genetic testing. They must be clinically stable at the time of recruitment (as assessed by the treating physician) and have a history of at least one admission to hospital to receive intravenous antibiotics over the preceding 24 months.

Exclusion criteria include: 1) currently participating in another clinical trial (excluding observational studies); 2) pneumothorax or lung surgery within the previous 3 months or eye surgery (e.g. cataract operation) in the previous 4 weeks (since these factors prevent measurement of spirometry); 3) airway infection with *Burkholderia cenocepacia* or *Mycobacterium abscessus* at the time of recruitment; 4) previous lung transplantation procedure.

### Sample size

Estimating the mean number of hospital inpatient days is 42 for those receiving standard care and 36 for those receiving monitoring, assuming a standard deviation of 10 and 40 patients per arm, approximate 95% confidence intervals for these estimates would be (39, 45) and (33, 39) respectively. To allow for patient drop out a total of 100 patients will be recruited (50 patients in each group).

### Recruitment and randomisation

**Fig. 1:**
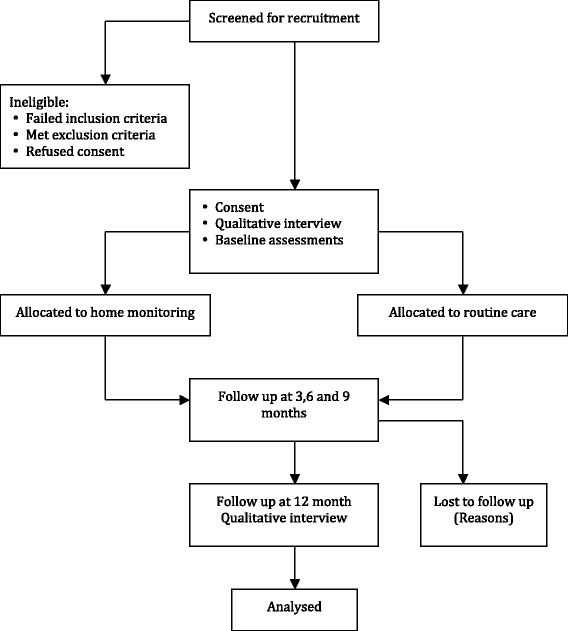
Flow of patients through the study

The flow of participants through the study will reflect the recommendations from the Consolidated Standards of Reporting Trials statement [16] and is outlined in Fig. 1. Participants will receive written and verbal information explaining the study and written consent will be obtained from all participants. West Midlands (Solihull) Research Ethics Committee (12/WM/0379) approved the study, Heart of England NHS Foundation Trust is the trial sponsor and the protocol is registered with Clinicaltrials.gov (NCT02994706).

Participants will be screened from the West Midlands Adult CF Centre database by the study investigators. If eligible, the study investigators will approach patients when they are attending routine clinic visits or during inpatient admissions to explain the study and if they are potentially interested they will be given the Participant Information Sheet. Having had time to consider the study and have any questions answered to their satisfaction, patients still keen to take part will be asked to provide written informed consent. It will be explained that they may withdraw from the study at any time.

At this point, baseline data will be obtained and the participant will be asked to take part in a semi-structured qualitative interview to assess the patient experience of their care to date and their expectations of participating in the study. Within strata, participants will then be randomly assigned to receive either home monitoring or routine care. The strata are based on the number of hospital admissions to receive intravenous antibiotics over the preceding 24 months, separated into four strata: ‘1–2 admissions’, ‘3–4 admissions’, ‘5–6 admissions’ and ‘more than seven admissions’. The randomisation will be completed by the selection of randomly ordered sealed opaque envelopes, with randomisation having been performed by a clerical member of the CF team with no knowledge of the study.

## Interventions

### Routine care group

These participants will continue to receive routine CF care, including regular outpatient clinic appointments and inpatient admissions if required.

### Home monitoring group

Participants randomized to receive home monitoring will be provided with a Bluetooth-enabled digital spirometer and a ‘Microsoft Windows Mobile’ enabled mobile phone. Participants will be shown how to use the equipment before going home. Participants will be asked to record their FEV_1_ using a digital spirometer twice weekly (Monday and Thursday), which will take around 5 min to complete. Participants will also record their symptoms twice weekly (Monday and Thursday) on the mobile phone by completing the Cystic Fibrosis Respiratory Symptom Diary – Chronic Respiratory Infection Symptom Score (CFRSD-CRISS) [5]. This is a validated disease-specific tool (consisting of 16 questions) designed to detect symptoms of pulmonary exacerbations in CF, taking around 5 min to complete.

Data regarding lung function and symptoms will be automatically transmitted via a secure encrypted wireless connection and the research team will be able to access this data instantaneously on a specially designed website. The mean values of the first 2 weeks’ data will be used to determine the participants’ baseline levels for FEV_1_ and CFRSD-CRISS during the run-in phase. Following this initial 2-week period, the CF team will receive an email alert if participants’ FEV_1_ or symptoms deteriorate below a set threshold. The criteria that will trigger an email alert are one of: 1) a drop in FEV_1_ of 10% or more from baseline; 2) total score of the CFRSD-CRISS worsening by more than three points from baseline. Following an email alert, the CF team will contact the participant within 24 h to discuss their symptoms. Based on this discussion, the research team will advise the participant on the best management. If the participant needs to be assessed in the outpatient clinic, we will arrange the next available convenient appointment. Depending on the clinical assessment, participants will receive a course of antibiotics, either oral or intravenous depending on the severity of symptoms, sputum microbiology and patient choice. If prescribed antibiotics, we will document whether these were prescribed for a protocol-defined pulmonary exacerbation (see definition below).

To minimize lack of adherence to home monitoring, we will re-enforce the importance of adhering to the study protocol at each study visit. The system will alert the research team if any participant fails to record symptoms and spirometry data. If this occurs, we will call the participant to check if they are having any problems with the equipment and that they are willing to proceed with the study. If there are any difficulties with using the equipment, we will explain how to resolve this issue. If any participant fails to submit data for more than 20% of occasions over any 28-day period we will advise them that they risk being withdrawn from the study. If there was a good reason for them not to submit data (such as a foreign holiday of which we were not aware) we will remind them to inform us if they will not be able to submit data. If the lack of data was due to non-adherence to the study protocol and if the participant fails to submit data for more than 20% of a subsequent 28-day period they will be withdrawn from the study. If the participant does not wish to proceed with the study at any stage they will be withdrawn and this will have no adverse effect on their routine care.

In addition to recording their symptoms and spirometry, subjects receiving home monitoring will be asked to test a urine sample once every week and send a urine sample in a prepaid envelope. These samples will be batched and subsequently analysed for biomarker analysis and this data will not be used by the research team during the course of this study.

## Outcome measures

All outcome measures will be recorded at baseline, 3, 6, 9 and 12 months, except for the semi-structured qualitative interview, which will be conducted at baseline and at 12 months (Fig. 1). Baseline data collection will include age, gender, weight (kg), body mass index (kg/m^2^), spirometry (FEV_1_, forced vital capacity (FVC)), concomitant medications, co-morbidities and health economic data. The number of courses of intravenous antibiotics and hospital admissions per year in the previous 2 years will also be recorded.

### Primary outcomes measures

The primary outcome measures are number of hospital inpatient bed days and health-related quality of life.

- **Hospital inpatient days** will be defined by number of complete 24-h periods between admission and discharge.
- **Health-related quality of life** will be measured by the Cystic Fibrosis Questionnaire – Revised (CFQ-R), a validated disease-specific health related quality of life questionnaire [17], which consists of 49 self-reported items within 12 domains: 1) physical functioning (8 items); 2) vitality (4 items); 3) emotional functioning (5 items); 4) eating disturbances (3 items); 5) treatment burden (3 items); 6) general health perception (3 items); 7) social functioning (6 items); 8) body image (3 items); 9) role limitations (4 items); 10) weight problems (1 item); 11) respiratory symptoms (6 items); and 12) digestive symptoms (3 items). Answers are reported on a four-point scale rating frequency, difficulty, or truth and the scores range from 0 to 100, with a higher score indicating better quality of life.

### Secondary outcome measures

- **Antibiotic requirements** will be measured by total number of completed days on oral and intravenous antibiotics.
- **Protocol defined pulmonary exacerbations** will be recorded using the modified Fuchs criteria [18], which requires the presence of 4 or more of the following criteria:
  · Change in sputum
  · New or increased haemoptysis
  · Increased cough
  · Increased dyspnoea
  · Malaise, fatigue, or lethargy
  · Temperature above 38 °C
  · Anorexia or weight loss
  · Sinus pain or tenderness
  · Change in sinus discharge
  · Change in physical examination of the chest
  · Decrease in pulmonary function by 10%
  · Radiographic changes indicative of pulmonary infection
- **Spirometry (FEV**_**1**_ **, FVC)** will be performed using a digital Vitalograph spirometer in the CF outpatient clinic in accordance with the American Thoracic Society guidelines [19]. The highest value for FEV_1_ and FVC obtained from three reproducible trials will be recorded and compared to predicted normal values.
- **Body weight** (kg) will be measured using digital scales.
- **Body mass index (BMI)** (kg/m^2^) will be measured using body weight and height recorded using a wall-mounted tape measure.
- **Anxiety and depression** will be measured by the Hospital Anxiety and Depression Scale (HADS) [20]. This questionnaire is designed to detect and measure the severity of anxiety and depression. It consists of a series of 14 statements, with responses based on a 4-point Likert scale. The HADS is self-administered, with a higher score being indicative of greater anxiety or depression.
- **Health economics analysis** will be conducted from a NHS and patient perspective. Participants will be asked to record any costs related to their care throughout the study, including the cost of visiting the hospital for outpatient visits (such as travel and parking costs) and loss of pay due to time off work. Participants will be asked to complete the EuroQol 5 dimensions (EQ-5D-5L), a generic health status questionnaire [21] and ICEpop CAPability measure for Adults (ICECAP-A), a generic measure of capability [22] at baseline, 3, 6, 9 and 12-month study visits. The researchers will record the costs associated with caring for each participant (such as costs of outpatient visits, inpatient admissions and staff time) and the cost of conducting the study (including staff costs and costs of the home monitoring equipment).

## Qualitative analysis

The qualitative aspects of the trial are a vital component in allowing us to better understand the context in which the trial takes place from the patient perspective. Participants will be invited to take part in semi-structured interviews at baseline and 12-months. These will help us to understand the reasons for patient participation, the contexts in which the trial takes place and will support interpretation of the findings (e.g. differential uptake or use of home-monitoring, reasons for patient withdrawal).

Topics of inquiry at baseline will include how individuals experience, understand and make decisions about their symptoms, health-care needs and service use. Participants’ expectations for home-monitoring will also be explored. Follow-up interviews will be undertaken with the intervention group to explore their experiences of using home-monitoring, including issues around acceptability and sustainability. This will include questions to explore the technical aspects of home-monitoring (e.g. ease of use), behavioural issues (e.g. perceived costs and benefits, necessary skill sets) and educational issues (e.g. adequacy of information and training). Suggestions for further improvement will also be sought. Where appropriate, follow-up interviews will examine reasons for study withdrawal.

### Statistical and data analysis

The outcomes listed will be summarized by group and 95% confidence intervals for these estimates will be calculated. Exploratory analyses will include basic tests to investigate differences between the intervention and control group, t-tests or chi-squared tests (or non-parametric alternative as appropriate). Cost-effectiveness will be assessed by calculating cost per reduction in hospital inpatient bed days and cost per quality-adjusted life year (QALYs) gained and appropriate sensitivity analyses will be conducted. Interview transcripts will be imported into NViVo and analysed using directed qualitative content analysis [23] to interpret meaning from the interview data in relation to the topics of inquiry, whilst allowing themes to emerge inductively from the data. To optimise rigour, data will be organized using the Framework method [24] and analysed as part of team approach, with an audit trail kept throughout. The qualitative and quantitative findings will be integrated using established methods [25] as part of the final analysis to allow us to interpret, understand and explain the trial findings.

### Safety

The Trial Management Group (TMG) will be responsible for day-to-day running of the trial and will meet on a monthly basis to ensure that the study is running smoothly. The Trial Steering Committee (TSC) will meet every 3 months to ensure that the study is run ethically. The Data Monitoring and Ethics Committee (DMEC) will meet every 3 months and its responsibilities will include: ensuring that the trial is recruiting subjects to schedule and assessing any adverse events associated with home monitoring. The DMEC can stop the trial early if felt necessary and will report to the Trial Steering Committee after every meeting. The TMG, TSC and DMEC will function independently of the sponsor and funding bodies.

## Discussion

People with CF typically suffer a progressive decline in lung function, resulting in premature mortality, most commonly due to respiratory failure [3]. Patients with more frequent exacerbations experience a more rapid decline in lung function and have a worse prognosis [26]. In 25% of pulmonary exacerbations, the patient suffers a permanent and irrecoverable loss of lung function [27]. Strategies aiming to prevent and/or minimize the impact of exacerbations are therefore vital for improving patient outcomes. Reducing hospital admissions by detecting early pulmonary exacerbations has the potential not only to improve lung health, but also to improve quality of life and reduce expenditure from the patient and healthcare provider perspective. However, there are also potential adverse effects of home monitoring, including increased anxiety caused by increased patient awareness of any deterioration in health status. This is the first randomized controlled study to assess the effect of home monitoring on relevant health outcomes combined with the health economic impact and a robust assessment of the patient experience.

The results of this study will allow people with CF and their carers, as well as those funding CF care, to fully evaluate the effect of home monitoring. This is particularly relevant, since as the survival of people with CF continues to improve, the feasibility of providing the current standard model of regular face-to-face CF clinical encounters becomes more challenging. Constraints on healthcare budgets worldwide make the provision of additional resources, including additional staff and new CF centres less realistic. Novel means of using technology to improve the interaction between people with CF and their MDT provides a potential solution to this increasingly difficult conundrum. Remote monitoring also potentially reduces the requirement for hospital visits and therefore could reduce opportunities for cross-infection, although this must be balanced against the theoretical benefits of regular direct patient contact with the MDT. Importantly, this study will examine the effect of home monitoring on psychological outcomes and the patient experience, assessing potential adverse effects such as increased anxiety, as well as potential benefits of patients feeling more empowered by the greater knowledge of their own health status.

## Abbreviations

BMI: Body mass index
CF: Cystic fibrosis
CFQ-R: Cystic fibrosis questionnaire-revised
CFRSD-CRISS: Cystic fibrosis respiratory symptom diary – chronic respiratory infection symptom score
CFTR: Cystic fibrosis transmembrane conductance regulator
DMEC: Data Monitoring and Ethics Committee
EQ-5D-5L: EuroQol 5 dimensions
FEV_1_: Forced expiratory volume in one second
FVC: Forced vital capacity
HADS: Hospital anxiety and depression scale
ICECAP-A: ICEpop CAPability measure for Adults
MDT: Multi-disciplinary team
NHS: National Health Service
NIHR: National Institute for Health Research
QALY’s: Quality-adjusted life years
RCT: Randomised controlled trial
TMG: Trial Management Group
TSC: Trial Steering Committee
